# A Review of The Society for Assisted Reproductive Technology Embryo Grading System and Proposed Modification

**DOI:** 10.22074/ijfs.2016.4956

**Published:** 2016-06-01

**Authors:** Amjad Hossain, John Phelps, Ashok Agarwal, Eduardo Sanz, Maha Mahadevan

**Affiliations:** 1 Division of Reproductive Endocrinology and Infertility, Department of Obstetrics and Gynecology, The University of Texas Medical Branch at Galveston, Galveston, Texas, USA; 2 Department of Urology, Cleveland Clinic Foundation, Cleveland, OH, USA; 3 Center for Reproductive Health, Crest Hill, IL, USA; 4 Division of Reproductive Endocrinology and Infertility, Department of Obstetrics and Gynecology, University of Arkansas for Medical Sciences, Little Rock, AR, USA

**Keywords:** Embryo, SART, Grading, Transfer

## Abstract

The Society for Assisted Reproductive Technology (SART) method of embryo grad- ing is unique, simple, and widely practiced, and its use has been mandatory for SART membership programs since 2010. Developed by SART in 2006, the current embryo grading system categories, “good, fair, and poor,” are limited because they do not describe the best 1-2 embryos in the interest of keeping pace with the shift in clinical practice to be more selective and to transfer fewer embryos. This inspired us to conduct a review on the SART embryo grading system.

In this retrospective study, the literature on evaluation of human embryo quality in gen- eral, and the SART method of evaluation in particular, were reviewed for the period of 2000 to 2014. A multifaceted search pertaining to methods of embryo grading and trans- fer using a combination of relevant terms [embryo, mammalian, embryo transfer, grade, grading, morphology, biomarkers, SART, and *in vitro* fertilization (IVF)] was performed. The inclusion and exclusion in this review were dictated by the aim and scope of the study. Two investigators independently assessed the studies and extracted information. A total of 61 articles were reviewed.

Very few studies have evaluated the efficacy of the SART embryo grading method. The present study suggests the necessity for revision of the current SART grading system. The system, as it is now, lacks criteria for describing the cohort specific best embryo and thus is of limited use in single embryo transfer. The study foresees heightened descriptive efficiency of the SART system by implementing the proposed changes.

Strengths and weaknesses of the SART embryo grading were identified. Ideas for selecting the best cohort-specific embryo have been discussed, which may trigger methodological improvement in SART and other embryo grading systems.

## Introduction

Embryo selection for embryo transfer (ET) is a crucial step of *in vitro* fertilization (IVF). Selecting the best embryo for achieving pregnancy from an embryo cohort has been a challenge for embryologists (1). In the early use of IVF for infertility treatment, morphological assessment of embryo quality was the method for choosing embryos and remains the mainstay of embryo selection today (1,3), but different morphological methods for grading IVF-generated embryos have been developed over time (4,12). Recently, biochemical and time-lapse analyses of embryo quality have been under investigation, but they are not yet fully ready for clinical application (13,16). Information about the efficiency and usefulness of these grading methods is important for improving ET success. 

Embryologists also recognize the necessity of developing a unifying standard method of grading embryos (17,19). The European Society of Human Reproduction and Embryology (ESHRE) is working to develop one such unifying embryo grading method (17,18,20,21). Some European countries such as the United Kingdom and Spain have already started to utilize a national standardized grading method (19,22,23). Likewise, embryologists in the United States under the banner of the Society for Assisted Reproductive Technology (SART) took the initiative to establish a uniform embryo grading method (1,24,25). The SART task force devised a grading system, applying a 3-point grading scale of “good, fair, and poor” in 2006 (24,25). The present study is a review of the current SART 3-point embryo grading method. The objective of this review was to find whether the SART method is fulfilling embryologists’ needs in selecting embryos for transfer. The review makes some suggestions which we believe will improve the SART embryo grading method’s usefulness for selecting the best embryo(s) for transfer. 

## Materials and Methods

**Fig.1:**
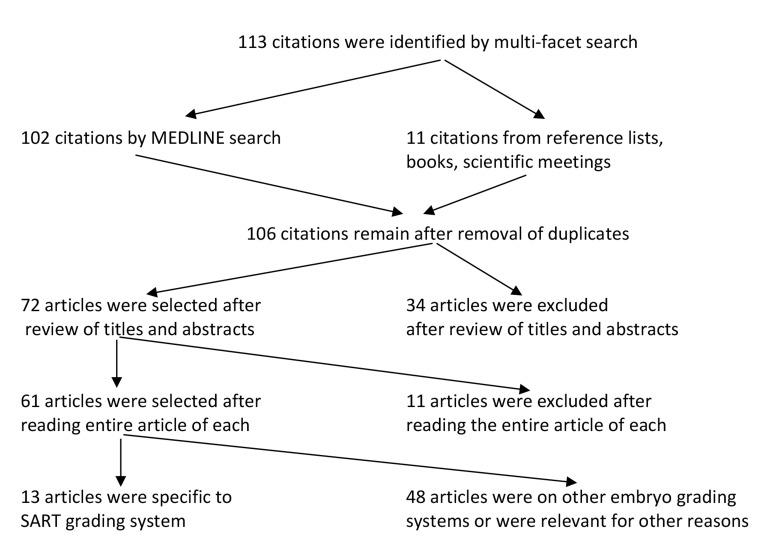
Flow chart showing selection and exclusion of articles in the systematic review. SART; Society for Assisted Reproductive Technology.

In this retrospective study, a review of the literature relevant to SART embryo grading system was conducted to assess its strengths and limitations. Information on evaluation of human embryo quality in general, and the SART method of evaluation in particular, was used. Several strategies were adopted to identify the pertinent articles. First, a multifaceted search performed for the period of 2000 to 2014 generated a total of 113 citations (Fig .1). The search utilized combinations of the following terms and subject headings: embryo, mammalian, ET, grade, grading, morphology, morphological parameters, biomarkers, SART, and IVF. Special emphasis was given to articles dealing with the efficiency of the SART grading system. Reference lists of relevant articles were searched manually to find additional reports which led us to select several articles prior to 2000. Proceedings of selected scientific meetings, book chapters, and monographs on embryo assessment were also reviewed. Articles found not relevant to the aim and scope of the present study were excluded from the review. Articles on the other embryo grading methods were included only if found pertinent to the scope of the study. Some of the search-generated items were excluded stepwise from the study if they were i. Duplicates (n=7) or ii. Irrelevant after reading the title and abstract (n=34) or the entire article (n=11). 

Institutional review board approval was not requested, as this was a review of published literature and not human research. 

## Results

### Characteristics of the studies retrieved and reviewed

The literature search had 2 components. The first component, which specifically focused on the SART grading method, produced 22 articles, of which 9 were not relevant to the objective of the study. The review findings of the remaining 13 articles are shown below under the section “Synopsis of the SART grading system”. Two authors (A.H. and M.M.) independently reviewed the articles and reached similar conclusions. Another 48 articles covering other grading methods and advances in IVF technologies, specifically those that had an association with embryo evaluation, comprised the second component of the search. This second set of 48 articles was reviewed, and the extracted information was collated with the first set (13 articles) to prepare the other sections of the manuscript (Fig .1). 

### Synopsis of the SART grading system

The review found that the SART members realized the necessity of developing a unifying standard method of grading embryos, and SART established a task force to explore such a possibility (24). In 2005, the task force developed a 3-point grading system using “good, fair, and poor” as grades. Three preconditions-must be simple, must have a basis in scientific inquiry, and must be easily adoptable in laboratories-guided the SART scheme. The grading utilized morphologic features applicable to 3 growth phases: cleavage, morula, and blastocyst (24,25). Compared to other grading methods, the SART method was found to have 2 unique attributes. First, the SART system uses words, such as “good, fair, and poor,” as grades, while other methods apply alphabet letters (A/a, B/b, C/c) and numerals (1/I, 2/II, 3/III) or their combinations as grades (1,17,18,24,26). Second, implementation of the SART grading system is endorsed by the nationally recognized organization that created it, while the majority of grading methods lack the advantage of being supported by a professional organization (18,24,26). 

The voluntary collection of embryo data employing the SART method began in 2006 and became mandatory in 2010 (24,25). The task force claimed an association between implantation and SART grades based on the initial set of SART embryo data. This relationship of SART grades and implantation was first reported at the 2009 American Society of Reproductive Medicine (ASRM) meeting and then in a number of journal articles (27,30). In the consensus workshop on embryo assessment sponsored by ALPHA scientists (an organization of scientists in reproduction) and ESHRE, a member of the SART task force made a presentation that highlighted the SART’s stand on standardized embryo grading (18). 

The Centers for Disease Control (CDC) has been responsible for publishing the SART embryo data since 2009 (31,33). The American Association of Bioanalysts (AAB) implemented a proficiency test, based on the SART method of grading, to standardize the grading skills of embryologists (34). Both the CDC and AAB remain committed to sharing the SART embryo grading outcomes with the public (31,32,34). Apart from those conducted by SART, CDC, and AAB, there were no evaluation studies, clinical trials, comparative analyses, or review studies on the SART grading system. The only studies outside of SART that made comments on the SART system were those of our group (35,36). Our study found SART grading applicable to all developmental stages from oocyte to blastocyst. 

### Limitations of the SART grading system and potential resolution

The SART system sorts the embryos of a cohort into 3 groups: good, fair, or poor (24,25). Since many IVF procedures produce a large number of embryos, obtaining several good embryos in each cohort is likely, and the same is true for the fair and poor categories (31,36,37). The dilemma, however, is determining which good embryo(s) to select for ET when several of the same grade are in the pool. 

The SART system does not have any provision for further discriminating the single best embryo from the available good embryos (24,25). Secondly, the SART method selects embryos based on static observation (1,24). This type of single snapshot examination may miss or overlook in-depth details, making the grading insufficient (38,41). 

**Table 1:** Potential upgrades for SART embryo grading method


Current SART grading method		Proposed changes in the SART grading method				
		Option 1			Option 2	
Existing grades	Number of embryo in the grade	Possible grades	Number of embryo in the grade	Embryo ranking in the grade	Possible grades	Number of embryo in the grade

Good	0 to M	Good	0 to M	R1, R2, R3, etc.	Best	0 to 1
Fair	0 to M	Fair	0 to M	R1, R2, R3, etc.	Better	0 to 1
Poor	1 to M	Poor	1 to M	R1, R2, R3, etc.	Good	0 to M
					Fair	0 to M
					Poor	1 to M


SART; Society for Assisted Reproductive Technology, M; Stands for multiple and R1, R2, R3, etc.; Represent rank 1, rank 2, and rank 3, etc., respectively.

Recent publications demonstrate that new knowledge and technological advancements that have occurred in the field, particularly in the assessment of embryo viability and implantation, are powerful enough for refining SART’s embryo selection strategy to overcome the challenge of embryo selection for ET (6, 42-49). Specifically, knowledge on sequential assessment, time-lapse monitoring, and profiling by “-omics” technology has grown significantly and shows great promise to add a new dimension to the embryo evaluation (7, 50-57). In addition to this literature-based projection, we have come up with specific ideas of our own to make the SART system a better fit to tackle the challenge of embryo selection for transfer (Table 1). In our proposal, we advocate for 2 different upgrades to the SART grading method (Table 1).

## Discussion

The SART grading system was based on 3 preconditions: must be simple, must have a basis in scientific inquiry, and must be easily adoptable in laboratories. Such preconditions were imposed for better standardization and easy execution of the system globally. The goal apparently has been achieved as SART grading became one of the most widely practiced grading methods.

In the era of highly efficient ovulation induc- tion, yields of multiple embryos in all 3 SART grades-good, fair, and poor-became common (32, 33, 36). The SART method classifies the embryos into 3 broad groups instead of selecting the best embryo for ET. By identifying embryos as good, fair, and poor, the SART system prepares a list of transfer-suitable embryos, not a rank-ordered list of embryo(s) for transfer. Ideally, the number of embryos for ET should be narrowed down to 1 embryo (32, 58, 59)-the best in the cohort-which is not achieved using the SART system. 

Our vision of the SART upgrade has been briefly outlined in Table 1. It presents 2 alternate sugges- tions to overcome the above indicated limitations of the SART system in embryo selection for ET. This proposal provides a guiding principle to rank a sequential list of embryos in the cohort. In op- tion 1 of the proposal (Table 1), we suggest grad- ing the embryos as “good, fair, and poor,” as it is currently done by the SART method; however, we recommend adding a second tier of ranking for the graded embryos. For example, in the event of ET, the embryos in the “good” group should be ranked further for selection for ET. If the “good” group has no embryo or has an insufficient num- ber of embryos, the embryos of the lower group should be ranked for ET. The target is to find the best embryo in the cohort. In the alternate plan (option 2), the SART system could be expanded to 5 grades instead of the current 3. Increasing the number of grades from 3 to 5 and simultaneously restricting the number of embryos to 1 in the top 2 grades (best and better) would compel the em- bryologist to serially tag the embryos, particularly the top 2. Emphasis is placed on 2 embryos be- cause 1-2 embryos are commonly used in ET (21, 32, 33, 37). In either plan (option 1 or option 2), in lieu of the one-time evaluation, the cumulative grade obtained by sequential monitoring, manual or electronic, should be favored for individual- izing the cohort-specific embryos. The SART method utilizes a set of parameters (cell number, fragmentation, and symmetry) for grading the cleaving embryos and another set of parameters (expansion, inner cell mass [ICM], and trophectoderm [TE]) for blastocyst grading (24,25). Many other studies, including our own, whose primary focus were embryo grading, found the following growth phase-specific morphological parameters ideal for embryo evaluation: zona pellucida (ZP), perivitelline space (PS), ooplasm, and polar body (PB) for oocyte; ZP, PS, pronucleus, and cytoplasm for zygote; number, quality, symmetry, fragmentation, and compaction of blastomeres in the cleaving embryos; and size (expansion), ICM, and TE for blastocysts (3,4,6,9,10,12,18,23,36,39). In our proposed upgrade, we emphasize continuous monitoring of the cohort members in the pool by employing the above mentioned growth phase-specific morphological parameters so that the cohort members can be ranked reflecting the differences in their quality, specifically their vigor and implantation potential. Although time-lapse and “-omics” technologies may have advantages in monitoring and ranking the embryos, many laboratories lack these advance technologies. These laboratories have to sharpen their embryo ranking skills based on the methodological resources available to them. No matter what method a laboratory applies in evaluation of embryos, conventional or advanced, the primary goal-ranking the embryos in the cohort-can eventually be achieved by the embryologist’s embryo monitoring skills. Based on this optimism, we suggest embryo ranking in both of our proposed upgrade options. Embryo selection for ET will hopefully be better served by the proposed changes in SART grading simply because they require the embryologist to rank the embryos in the respective cohort. Future studies will cultivate this important concept of ranking embryos to develop a comprehensive upgrade plan for the SART system. 

Selecting the best embryo for transfer could perhaps be achieved if the SART system would rank the embryos the way students in a class are ranked based on cumulative assessments. A successful embryo ranking would improve the ability to assess the relative vivacity and implantation potential of individual embryos within a cohort, perhaps lessening the need to transfer more than 1 embryo. With accurate embryo ranking, it is not unreasonable to assume that if the best ranked embryo cannot result in implantation, the lower-ranked embryos will be less likely to implant in an equitable uterine environment. Thus, if validated embryo ranking can be achieved, the practice of transferring lower quality embryos with the thought of improving pregnancy rates may become less common. 

The primary aim of embryo ranking should be to discriminate the viability and implantation potential among embryos of a cohort (6,42,44,45,48,53,55). Two concepts are becoming increasingly evident from recent studies: i. Improved understanding of embryo implantation is necessary to enhance success in selecting the best embryo, (1,5,12,18,40,52,54,57,60) and ii. Sequential assessment has an advantage over single assessment in finding the best embryo (8,11,38,42,51). In addition, recent studies also suggest that advanced high-technology IVF techniques, compared to conventional IVF, are more effective for investigating the viability and implantation potential of embryos (3,12,26,46,49). In the near future, 2 of these advanced IVF techniques, 1 using timelapse monitoring technology (3,16,46,49,57,61) and the other using “-omics” technology, (14,43,48,52,54) may become capable of efficiently discriminating the embryo viability and implantation. 

## Conclusion

The present study shows the strengths and weaknesses of the SART grading system. SART grading was established for the noble mission of developing a unifying standard method of grading human embryo. It has helped immensely in standardizing grading systems among clinics. The joint effort of SART and AAB in developing an embryo gradingrelated proficiency test homogenizes the embryo grading skills of the embryologists. However, with the shift in clinical practice to transfer fewer embryos, the current SART system falls short in fulfilling its ultimate goal-selecting the right embryo for ET. Apart from SART itself, we found no evaluation studies or clinical trials on the efficiency of the SART grading method. The authors of this manuscript humbly suggest that the time for upgrading the current SART grading system to include a more descriptive ranking of embryos is due. Our proposed additions to the current SART grading system are simple and can be implemented by any IVF laboratory without the need for additional equipment. Moreover, it would better permit a descriptive process to delineate the best embryos Proposed SART Embryo Grading System Modifications 146 for transfer rather than a cohort of embryos. 
